# Convergence of nanotechnology and artificial intelligence in the fight against liver cancer: a comprehensive review

**DOI:** 10.1007/s12672-025-01821-y

**Published:** 2025-01-22

**Authors:** Manjusha Bhange, Darshan Telange

**Affiliations:** Department of Pharmaceutics, Datta Meghe College of Pharmacy, Datta Meghe Institute of Higher Education and Research (DU), Sawangi Meghe, Wardha, Maharashtra 442001 India

**Keywords:** Liver cancer, Nanotechnology, Artificial intelligence, Personalized medicine

## Abstract

Liver cancer is one of the most challenging malignancies, often associated with poor prognosis and limited treatment options. Recent advancements in nanotechnology and artificial intelligence (AI) have opened new frontiers in the fight against this disease. Nanotechnology enables precise, targeted drug delivery, enhancing the efficacy of therapeutics while minimizing off-target effects. Simultaneously, AI contributes to improved diagnostic accuracy, predictive modeling, and the development of personalized treatment strategies. This review explores the convergence of nanotechnology and AI in liver cancer treatment, evaluating current progress, identifying existing research gaps, and discussing future directions. We highlight how AI-powered algorithms can optimize nanocarrier design, facilitate real-time monitoring of treatment efficacy, and enhance clinical decision-making. By integrating AI with nanotechnology, clinicians can achieve more accurate patient stratification and treatment personalization, ultimately improving patient outcomes. This convergence holds significant promise for transforming liver cancer therapy into a more precise, individualized, and efficient process. However, data privacy, regulatory hurdles, and the need for large-scale clinical validation remain. Addressing these issues will be essential to fully realizing the potential of these technologies in oncology.

## Introduction

Liver cancer, particularly hepatocellular carcinoma (HCC), is among the most prevalent and deadly cancers globally. It ranks as the sixth most common cancer and the third leading cause of cancer-related deaths, with high incidence rates in regions such as East Asia and sub-Saharan Africa [1]. Major risk factors for liver cancer include chronic hepatitis B and C infections, cirrhosis due to excessive alcohol consumption, non-alcoholic fatty liver disease (NAFLD), and metabolic disorders [2]. Despite progress in diagnostic methods and treatment strategies, liver cancer continues to present significant therapeutic challenges, particularly due to its late-stage diagnosis, high recurrence rates, and complex tumor microenvironment [3].

One of the primary hurdles in liver cancer management is the difficulty of early detection. In many cases, diagnosis occurs at advanced stages, leading to limited treatment options and poor prognosis [4]. Traditional treatments such as surgery, liver transplantation, and chemotherapy are constrained by patients' overall health, liver function, and tumor aggressiveness. Furthermore, liver cancer is often resistant to chemotherapy and radiation, complicating treatment [5]. Even the advent of targeted therapies, such as sorafenib, has shown only modest improvements in patient outcomes [6].

The need for more effective treatment options has led to the exploration of innovative technologies, including nanotechnology and artificial intelligence (AI). These fields have demonstrated the potential to address key liver cancer diagnosis and treatment challenges. Nanotechnology allows precise drug delivery to cancer cells, while AI enhances diagnostic accuracy and personalizes treatments [7]. AI algorithms predict the ideal shape and size of nanoparticles to maximize tumor penetration. Research by Zhang et al. [66] demonstrated that rod-shaped nanoparticles designed using AI had superior tumor-targeting efficacy compared to their spherical counterparts. The shape having non-spherical nanoparticles (e.g., rod-shaped or disc shaped) exhibit better tumor accumulation and cellular uptake compared to spherical nanoparticles due to their unique flow dynamics and interaction with biological barriers. The nanoparticles ranging from 10 to 100 nm are ideal for passive tumor targeting through the enhanced permeability and retention (EPR) effect [8]. However, size must also account for systemic clearance mechanisms such as kidney filtration and macrophage uptake. The AI models, including deep learning frameworks and genetic algorithms, predict the ideal shape and size of nanoparticles for liver cancer therapy. These predictions are based on datasets encompassing tumor biology, nanoparticle interactions, and physiological parameters [9].

Zhang et al. [66] demonstrated the use of AI algorithms to design rod-shaped nanoparticles for liver cancer treatment. Their study revealed that rod-shaped nanoparticles had 25% higher penetration into tumor tissues compared to spherical nanoparticles. A similar study employed genetic algorithms to optimize the size of polymeric nanoparticles, resulting in enhanced drug accumulation in HCC tumors by 18% in preclinical models [10]. AI models simulate the behavior of nanoparticles in dynamic biological environments, such as blood flow or tumor interstitial fluid. AI ensures their structural stability and targeting efficiency by predicting how nanoparticles deform under shear stress or interact with extracellular matrices. Emerging AI tools, such as digital twins, could simulate the behavior of nanoparticles in virtual patient models, allowing researchers to test and refine designs before actual clinical trials. This approach would significantly reduce development costs and timelines [11].

### Nanotechnology in healthcare and liver cancer treatment

Nanotechnology, defined as the design, production, and application of materials at the nanometer scale, has transformative potential in healthcare. Nanotechnology is particularly beneficial in cancer treatment due to its ability to selectively target cancer cells, reducing damage to healthy tissues and enhancing drug efficacy [12]. Nanoparticles, ranging from 1 to 100 nm, can be engineered to deliver drugs directly to tumors, improving drug bioavailability and overcoming the limitations of traditional therapies [13]. Nanotechnology has emerged as a promising tool for diagnosis and treatment of liver cancer. Nanoparticles can be used as imaging agents, improving the early detection of liver tumors with enhanced contrast in imaging modalities such as MRI and CT [14]. Additionally, nanocarriers like liposomes, dendrimers, and polymeric nanoparticles are being developed to deliver drugs specifically to liver tumors, bypassing natural defense mechanisms. These nanocarriers can be functionalized to enhance targeting specificity, especially in targeting liver cancer’s unique tumor microenvironment [15]. Nanotechnology also facilitates the development of theranostics, which combines diagnostics and therapeutics into a single platform [16]. Theranostic nanoparticles enable the simultaneous delivery of therapeutic agents while providing real-time feedback on treatment efficacy, allowing for personalized and adaptive treatment strategies. This integration could significantly improve liver cancer treatment by ensuring therapy efficacy while minimizing unnecessary toxicity [17].

Moreover, nanotechnology has the potential to overcome multidrug resistance (MDR) in liver cancer, a common challenge in chemotherapy. MDR arises when cancer cells develop resistance to multiple drugs, making conventional therapies ineffective. Nanoparticles, particularly polymer-based ones, can help bypass these resistance mechanisms by directly delivering drugs into cancer cells [18].

### AI applications in diagnostics and imaging for liver cancer

AI has been a transformative force in various medical disciplines, especially in oncology, where it enhances diagnostic accuracy and improves patient care. AI applications in imaging technologies such as ultrasound, computed tomography (CT), and magnetic resonance imaging (MRI) have significantly impacted liver cancer diagnosis. These tools offer a more refined assessment of tumor size, location, and characteristics, which are crucial in staging liver cancer and planning treatments [19].

For liver cancer, AI-driven diagnostic systems analyze imaging data to detect tumors that might be overlooked by human radiologists. A study by Yasaka et al. [32] demonstrated how convolutional neural networks (CNNs), a type of deep learning algorithm, were able to detect liver tumors in CT scans with higher accuracy than traditional methods [20]. AI algorithms have also shown promise in differentiating between malignant and benign lesions, reducing the risk of unnecessary biopsies or surgeries. This ability to perform nuanced analysis is vital in liver cancer, where lesions often mimic benign growths, complicating diagnosis [21]. Incorporating AI into imaging also supports the identification of early-stage tumors, when treatment outcomes are more favorable. AI-powered imaging systems can analyze subtle differences in tissue density and texture that might be missed by the human eye, allowing for earlier detection and, potentially, curative treatments [22]. AI has also been utilized to optimize radiomic analysis, a process that extracts large amounts of quantitative features from medical images. The radiomics approach can detect micro-level features of the tumor that correlate with its molecular and genetic profile, which assists in making more personalized treatment decisions. These radiomic features, when combined with machine learning (ML) algorithms, provide predictive insights into how a tumor may behave, further enhancing the diagnostic accuracy and prognosis [23]. ML, a subset of AI, is particularly useful in predictive modeling in oncology. Predictive modeling focuses on forecasting disease progression, patient survival, and response to therapies. For liver cancer, ML models analyze a multitude of variables, including clinical, genetic, and imaging data, to generate predictions that guide personalized treatment [24]. For instance, ML algorithms can be trained on clinical datasets that include patient demographics, tumor characteristics, liver function, and treatment outcomes. These models can predict which patients are likely to benefit from surgical interventions, such as liver resection or transplantation, versus those who may respond better to systemic therapies, such as chemotherapy or immunotherapy [25]. Moreover, ML systems can assist in assessing the risk of postoperative complications, recurrence, or metastasis, all of which play critical roles in treatment planning. One breakthrough in predictive modeling involves using AI to predict treatment responses in patients undergoing targeted therapies or immunotherapies. HCC the most common form of liver cancer, is often treated with sorafenib, a kinase inhibitor [26]. However, not all patients respond equally to this therapy. ML models have been employed to identify biomarkers, such as gene expression profiles or specific mutations, that predict a patient’s likelihood of responding to sorafenib.

This level of personalized prediction also extends to liver cancer’s response to radiation therapy [27]. AI algorithms, leveraging large datasets of patient outcomes, can simulate various treatment plans and predict how a tumor might shrink or grow under different radiation doses. This ensures that radiation is delivered precisely and effectively, reducing harm to healthy liver tissues [28].

### AI algorithms for personalized treatment planning

AI’s role in liver cancer treatment extends beyond diagnostics and predictive modeling into personalized treatment planning. Personalized medicine is critical for liver cancer is due to the complex interaction between the tumor, the liver’s regenerative capacity, and underlying liver diseases, such as cirrhosis or hepatitis [29].

AI-powered treatment planning uses algorithms that synthesize large datasets comprising patient health records, genomic data, tumor biology, and drug response information. This allows for a tailored treatment approach where therapies are chosen based on the patient’s specific cancer subtype and overall health profile. For example, ML algorithms can recommend the best systemic therapies for patients with HCC based on their gene expression patterns or suggest which patients would benefit from combination therapies, such as chemotherapy with immunotherapy [30]. A significant area where AI has shown potential is in optimizing treatment regimens for patients undergoing liver transplantation. AI models can predict graft survival, risk of recurrence, and postoperative complications, all of which are critical in determining eligibility and timing for liver transplantation. Moreover, AI algorithms are being used to assist in selecting patients for clinical trials, a vital step in developing new liver cancer treatments. Using predictive modeling, AI systems can identify patients who are most likely to respond positively to experimental treatments based on their genetic and clinical profiles. This improves the efficiency of clinical trials and accelerates the approval of new therapies [31, 32].

### Integration of AI with robotic surgeries and real-time monitoring

Sun et al. [2020] mentioned that integrating AI into robotic surgeries represents a significant advancement in liver cancer treatment. Robotic-assisted surgeries provide greater precision, flexibility, and control than traditional open or laparoscopic surgeries, which are particularly beneficial when operating on complex structures like the liver. AI enhances these procedures by providing real-time guidance during surgery [33].

For liver cancer, AI-guided robotic systems can precisely target tumors, even those located in challenging areas of the liver. These systems rely on AI algorithms to process real-time imaging data and adjust surgical tools dynamically. For instance, AI can help guide a robotic arm to remove a tumor while avoiding critical structures such as blood vessels or bile ducts [34]. This level of precision minimizes surgical complications and speeds up recovery times. Additionally, AI can be used in conjunction with nanotechnology to monitor treatment in real time. For example, AI algorithms can analyze real-time imaging data from nanoparticles used in drug delivery systems to track how well a liver tumor is responding to therapy. This provides clinicians with immediate feedback and allows for rapid adjustments in treatment [35].

The application of AI in liver cancer treatment represents a transformative step toward more precise, personalized, and effective therapies. AI's integration into oncology holds immense promise, from improving diagnostics and predictive modeling to enhancing treatment planning and robotic-assisted surgeries. While challenges such as data quality, algorithm transparency, and clinical adoption remain, ongoing advancements in AI technology are expected to significantly improve liver cancer patient outcomes in the coming years [36].

### Convergence of nanotechnology and AI in liver cancer

**Fig. 1:**
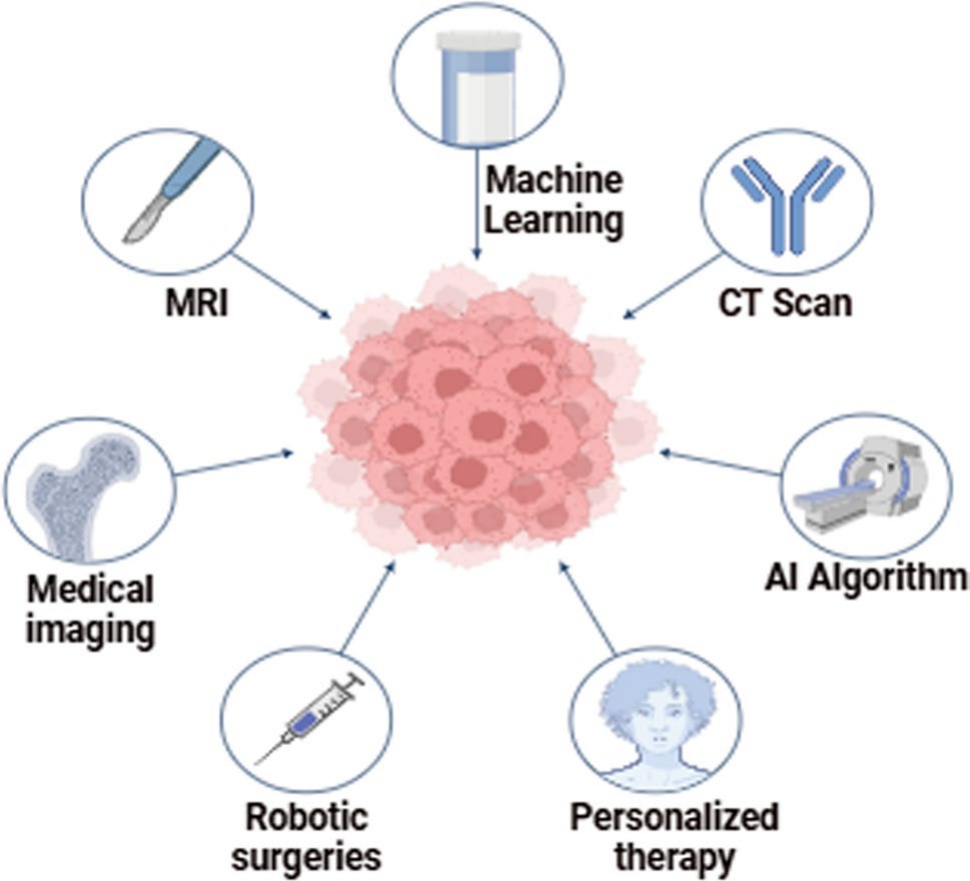
AI and technological advancements in liver cancer

Nanotechnology and AI are both advancing rapidly and reshaping various scientific fields, particularly in the treatment of liver cancer. The convergence of these two technologies promises to revolutionize diagnosis, drug delivery, and treatment by enhancing the precision, efficiency, and personalization of therapy [37]. This synergy optimizes the potential of nanotechnology by utilizing AI's computational power to design more effective nanocarriers, monitor drug release, and improve therapeutic outcomes. In this section, we will explore case studies and examples where both technologies are employed, AI-driven optimization of nanocarrier design, the role of AI in monitoring drug release and efficacy, and how precision medicine is enhanced through this collaboration [38]. Figure 1 illustrates the integration of advanced technologies like ML, AI Algorithms, and Medical Imaging in healthcare. These innovations enhance precision diagnostics support personalized therapies, and enable robotic surgeries, revolutionizing patient care.

## Real-life applications of AI and nanotechnology in liver cancer

AI-driven nanotechnology has shown promising results in the following areas:

### Smart nanocarriers

Liposomes, dendrimers, and polymeric nanoparticles enhanced with AI algorithms are being developed for controlled drug release. For example, AI-optimized liposomes encapsulating sorafenib showed improved drug delivery efficiency in animal models of HCC. Smart nanocarriers, including liposomes, dendrimers, and polymeric nanoparticles, are at the forefront of targeted drug delivery systems. AI algorithms are critical in optimizing their design for controlled and precise drug release [39]. Liposomes are widely used for encapsulating chemotherapeutic agents due to their biocompatibility and ability to target specific tissues. AI-driven models analyze drug release kinetics, lipid compositions, and surface modifications to enhance delivery efficiency. The Sorafenib-Loaded Liposomes case studies were on AI algorithms optimized liposomal formulations of sorafenib, a first-line therapy for advanced liver cancer, by predicting the ideal lipid composition and particle size. In preclinical studies involving animal models of HCC, these AI-optimized liposomes demonstrated superior drug accumulation in tumor tissues, reduced systemic toxicity, and enhanced therapeutic outcomes [30, 40]. Polymeric Nanoparticles: Polymeric nanoparticles designed with AI assistance allow precise control over drug release profiles. ML models predict polymer degradation rates, ensuring sustained drug release over an extended period. For instance, nanoparticles composed of poly (lactic-co-glycolic acid) (PLGA) were enhanced using AI to encapsulate doxorubicin, showing a 30% increase in therapeutic efficacy against HCC cells in vitro. Dendrimers, with their highly branched structures, provide ample functionalization sites for drug molecules. AI-guided optimization of dendrimer surface chemistry has improved drug loading capacities and targeting efficiencies, particularly in challenging microenvironments like the liver [41].

### AI-guided imaging agents

Gold nanoparticles functionalized with AI-designed ligands enhance contrast in CT imaging, enabling precise tumor delineation. AI algorithms also optimize the design of quantum dots for fluorescence imaging of liver tumors. AI-Guided Imaging Agents for Enhanced Diagnostics. Accurate tumor visualization is crucial for diagnosing liver cancer, planning treatments, and monitoring therapeutic responses [42]. AI-guided imaging agents, such as gold nanoparticles and quantum dots, are revolutionizing diagnostic precision. Gold nanoparticles (AuNPs) are excellent contrast agents for CT imaging due to their high atomic number and biocompatibility. AI algorithms optimize the functionalization of these nanoparticles by targeting ligands, ensuring selective accumulation in liver tumors. An example is AI-designed ligands. AI was employed to design ligands that bind specifically to overexpressed biomarkers on HCC cells, such as glypican-3 (GPC3). In animal studies, these AI-optimized gold nanoparticles significantly enhanced CT imaging contrast, enabling precise tumor delineation and early detection of liver cancer [43, 44].

Quantum dots are nanoscale semiconductors that emit fluorescence when excited by light, making them valuable for tumor imaging. AI-driven design processes optimize their size, surface coatings, and emission spectra for liver cancer applications. Real-world Applications of quantum dots are identified as an optimal surface coating for quantum dots to minimize nonspecific binding and enhance the targeting of alpha-fetoprotein (AFP), a common liver cancer biomarker. This resulted in improved fluorescence imaging of liver tumors, aiding in early diagnosis and real-time surgical guidance [45]. Other things like Multimodal Imaging Agents in AI are also advancing the development of multimodal nanoparticles that combine CT, MRI, and fluorescence imaging capabilities. These agents offer comprehensive tumor profiling, providing detailed insights into tumor size, vascularization, and metabolic activity.

Smart nanocarriers, including liposomes, dendrimers, and polymeric nanoparticles, are at the forefront of targeted drug delivery systems. AI algorithms play a critical role in optimizing their design for controlled and precise drug release [46]. The AI-Optimized Liposomes are widely used for encapsulating chemotherapeutic agents due to their biocompatibility and ability to target specific tissues. AI-driven models analyze drug release kinetics, lipid compositions, and surface modifications to enhance delivery efficiency. The Case Study of Sorafenib-Loaded Liposomes is an AI algorithms optimized liposomal formulations of sorafenib, a first-line therapy for advanced liver cancer, by predicting the ideal lipid composition and particle size. In preclinical studies involving animal models of HCC, these AI-optimized liposomes demonstrated superior drug accumulation in tumor tissues, reduced systemic toxicity, and enhanced therapeutic outcomes [47].

The polymeric nanoparticles designed with AI assistance allow precise control over drug release profiles. ML models predict polymer degradation rates, ensuring sustained drug release over an extended period. For instance, nanoparticles composed of poly (lactic-co-glycolic acid) (PLGA) were enhanced using AI to encapsulate doxorubicin, showing a 30% increase in therapeutic efficacy against HCC cells in vitro [48].

The dendrimers, with their highly branched structures, provide ample functionalization sites for drug molecules. AI-guided optimization of dendrimer surface chemistry has improved drug loading capacities and targeting efficiencies, particularly in challenging microenvironments like the liver [49]. The lipid-based nanoparticles are one of the most widely studied and applied nanocarriers in liver cancer treatment. Liposomes, solid lipid nanoparticles (SLNs), and nanostructured lipid carriers (NLCs) are examples of lipid-based nanocarriers that have demonstrated potential in targeted drug delivery. These nanocarriers have biocompatible and biodegradable properties, making them suitable for clinical use [50]. Liposomes are spherical vesicles composed of a lipid bilayer, which can encapsulate both hydrophilic and hydrophobic drugs. Due to their biocompatibility, liposomes have been widely investigated for drug delivery in liver cancer. Liposomal formulations of chemotherapeutic agents, such as doxorubicin and paclitaxel, have been developed to improve drug accumulation in liver tumors. Additionally, liposomes can be functionalized with targeting ligands such as antibodies or peptides to enhance the active targeting of liver cancer cells [51]. Solid lipid nanoparticles (SLNs) consist of a solid lipid core stabilized by surfactants. SLNs offer advantages such as improved stability, controlled drug release, and protection of encapsulated drugs from degradation. They have been utilized to deliver drugs such as sorafenib and curcumin, both of which have shown the potential to inhibit liver cancer growth. SLNs have also been shown to improve drug bioavailability and reduce systemic side effects [52].

Nanostructured lipid carriers (NLCs) are second-generation lipid nanoparticles composed of a mixture of solid and liquid lipids. NLCs provide a more flexible and stable drug delivery system than SLNs. Their lipid matrix allows for higher drug loading, and their small size facilitates accumulation in the tumor microenvironment. NLCs have been investigated for the delivery of various anticancer agents, including doxorubicin, paclitaxel, and curcumin, for the treatment of liver cancer [53]. Polymeric nanoparticles have garnered attention in liver cancer treatment due to their versatility in drug delivery. These nanocarriers can be engineered from natural or synthetic polymers and are capable of controlled drug release, biodegradability, and high drug-loading capacity. Commonly used polymers include poly(lactic-co-glycolic acid) (PLGA), polyethylene glycol (PEG), chitosan, and polycaprolactone (PCL) [54]. The PLGA nanoparticles are widely studied due to their biodegradability and biocompatibility. PLGA-based nanoparticles have been used to deliver chemotherapeutic agents, such as doxorubicin and paclitaxel, to liver cancer cells. These nanoparticles can be functionalized with targeting moieties, such as folic acid or transferrin, to enhance selective drug delivery to cancer cells [55].

Chitosan-based nanoparticles are another promising polymeric delivery system. Chitosan, a natural polymer derived from chitin, is biocompatible, biodegradable, and possesses mucoadhesive properties. Chitosan nanoparticles have been utilized to deliver drugs and genes for the treatment of liver cancer. Studies have shown that chitosan nanoparticles improve drug solubility, enhance drug delivery to tumor cells, and reduce systemic toxicity [30].

PEGylation of nanoparticles refers to the attachment of PEG chains to the surface of nanoparticles. PEGylation increases the circulation time of nanoparticles by reducing their recognition and clearance by the reticuloendothelial system (RES). PEGylated nanoparticles have been shown to improve the therapeutic efficacy of drugs in liver cancer by enhancing their accumulation in tumor tissues [56].

Metal-based nanoparticles, such as gold, silver, and iron oxide nanoparticles, have shown significant potential in liver cancer treatment due to their unique optical, magnetic, and chemical properties. These nanoparticles can be used for both therapeutic and diagnostic purposes, often referred to as theranostics. Metal-based nanoparticles offer advantages in imaging, tumor targeting, and drug delivery [57]. Gold nanoparticles (AuNPs) are widely studied for their biocompatibility and ability to absorb and scatter light. AuNPs can be used for imaging liver cancer tumors, enabling early detection and precise tumor localization. Additionally, gold nanoparticles can be functionalized with targeting ligands and loaded with drugs or genes to deliver therapies directly to liver cancer cells [58]. Iron oxide nanoparticles (IONPs) are magnetic nanoparticles that can be used for both imaging and drug delivery. These nanoparticles have been employed in MRI for liver cancer diagnosis. In addition to imaging, iron oxide nanoparticles can be functionalized with therapeutic agents for targeted drug delivery. Their magnetic properties also enable hyperthermia treatment, where localized heating destroys cancer cells [59]. Silver nanoparticles (AgNPs) have demonstrated potent anticancer activity due to their ability to induce oxidative stress and apoptosis in cancer cells. AgNPs have been studied for their role in inhibiting liver cancer cell proliferation and tumor growth. However, concerns regarding the long-term toxicity of silver nanoparticles remain, necessitating further investigation [60].

Tumor targeting using nanotechnology can be classified into passive and active targeting mechanisms. Both approaches are employed to enhance the delivery of therapeutic agents to liver cancer cells while minimizing off-target effects. Passive targeting relies on the EPR effect, where nanoparticles passively accumulate in tumor tissues due to the leaky vasculature and poor lymphatic drainage of tumors. The EPR effect allows nanoparticles to concentrate in liver tumors, improving drug delivery without the need for specific targeting ligands [61]. Active targeting involves functionalizing nanoparticles with specific ligands that bind to overexpressed receptors on the surface of cancer cells. Commonly used targeting ligands include antibodies, peptides, and small molecules that recognize and bind to receptors such as folate receptors, transferrin receptors, and epidermal growth factor receptors (EGFR). Active targeting enhances the selectivity of drug delivery and improves therapeutic outcomes in liver cancer [62]. One of the most studied examples of active targeting in liver cancer is the use of *folic acid- functionalized nanoparticles*. Folic acid binds to folate receptors, which are overexpressed on the surface of many cancer cells, including liver cancer cells. Folic acid-functionalized nanoparticles have been used to deliver doxorubicin and other anticancer agents to liver cancer, improving therapeutic efficacy. Another active targeting strategy involves the use of *transferrin-functionalized nanoparticles*. Transferrin receptors are often upregulated in cancer cells due to their increased demand for iron. Nanoparticles functionalized with transferrin have shown promise in delivering anticancer drugs specifically to liver tumors [63, 64].

### Personalized nanosystems

AI integrates patient-specific genomic and proteomic data to design nanoparticles tailored to individual liver cancer profiles. For instance, AI has been used to develop lipid nanoparticles targeting mutant p53 proteins commonly found in liver cancer. The heterogeneity of liver cancer poses significant challenges for conventional therapies. AI-driven personalized nanosystems address this issue by integrating patient-specific genomic, proteomic, and clinical data to design tailored therapeutic solutions. AI models analyze large datasets of genetic mutations, protein expressions, and tumor microenvironment characteristics to design nanoparticles that specifically target liver cancer subtypes [65, 66]. The mutations in the p53 tumor suppressor gene are common in liver cancer. AI algorithms were used to develop lipid nanoparticles targeting mutant p53 proteins. These nanoparticles encapsulated siRNA specifically designed to silence mutant p53, restoring normal cell function. In preclinical models, this approach demonstrated selective tumor targeting and significant tumor regression [67]. AI integrates multi-omics data (genomics, transcriptomics, and proteomics) to identify novel biomarkers and therapeutic targets. This data is then used to design nanoparticles functionalized with ligands that bind to these targets. For example, AI-designed nanoparticles targeting liver cancer cells with overexpressed fibroblast growth factor receptors (FGFRs) showed a 50% increase in drug uptake compared to non-targeted systems [68]. AI-powered adaptive nanoplatforms can adjust their properties in response to the tumor microenvironment. For instance, pH-sensitive nanoparticles developed using AI release their drug payload only in the acidic environment of liver tumors, minimizing damage to healthy tissues. While AI-driven nanotechnology holds immense promise, challenges such as scalability, regulatory approval, and clinical translation remain. Addressing these issues requires interdisciplinary collaboration among AI researchers, nanotechnologists, and clinicians [69, 70]. Developing more robust AI models that integrate real-time feedback from clinical trials could accelerate the optimization of nanocarriers and imaging agents. Ensuring the safety and biocompatibility of AI-designed nanoparticles is critical for regulatory approval and clinical adoption. AI could assist in predicting long-term toxicity and biodistribution of nanoparticles, addressing key safety concerns. The ultimate goal is to create personalized treatment plans where AI designs and monitors nanoparticle-based therapies tailored to individual patients, paving the way for precision oncology [71]. Table 1 summarizes based on the provided application area, AI contributions with their outcomes.

**Table 1:** Real-life applications of AI and nanotechnology in liver cancer AI-driven nanotechnology

Application area	Specific nanotechnology	AI contribution	Examples/outcomes	References
Smart nanocarriers	Liposomes	Optimizing lipid composition and particle size for targeted drug delivery	AI-optimized sorafenib-loaded liposomes showed enhanced drug accumulation in tumors, reduced toxicity, and improved therapeutic outcomes in HCC models	[30, 39, 40, 46, 47]
	Polymeric nanoparticles	Predicting polymer degradation rates for sustained drug release	PLGA nanoparticles encapsulating doxorubicin achieved 30% higher efficacy in vitro against HCC cells	[41, 48, 54]
	Dendrimers	Enhancing drug loading and targeting efficiency via surface chemistry optimization	Improved drug loading and targeting efficiency in challenging environments like the liver	[41, 49]
AI-guided imaging agents	Gold nanoparticles (AuNPs)	Designing ligands for better tumor targeting	Enhanced CT imaging contrast with AI-designed ligands binding to biomarkers like glypican-3	[42–44, 58]
	Quantum dots	Optimizing size, surface coatings, and emission spectra	Improved fluorescence imaging targeting alpha-fetoprotein (AFP) for liver cancer detection and surgery guidance	[45]
	Multimodal nanoparticles	Combining CT, MRI, and fluorescence imaging	Comprehensive tumor profiling, assessing size, vascularization, and metabolic activity	[42, 46]
Personalized nanosystems	Lipid nanoparticles	Tailoring to specific mutations like p53 using genomic and proteomic data	Lipid nanoparticles targeting mutant p53 proteins achieved significant tumor regression in preclinical models	[65–67]
	Functionalized nanoparticles	Targeting specific liver cancer biomarkers (e.g., FGFRs)	AI-designed FGFR-targeted nanoparticles improved drug uptake by 50%	[68]
	Adaptive nanoplatforms	Developing pH-sensitive systems for controlled drug release	Nanoparticles released drugs selectively in acidic liver tumor environments	[69, 70]
Metal-based nanoparticles	Gold nanoparticles (AuNPs)	Functionalization for imaging and therapy	AuNPs used for liver cancer imaging and delivery of targeted drugs	[58]
	Iron oxide nanoparticles (IONPs)	Enhancing imaging and enabling hyperthermia therapy	Used for MRI diagnostics and localized hyperthermia treatment	[59]
	Silver nanoparticles (AgNPs)	Addressing oxidative stress and apoptosis induction	Shown to inhibit liver cancer growth but with concerns over long-term toxicity	[60]

## Research combining AI and nanotechnology in precision oncology

The convergence of AI and nanotechnology has ushered in a new era of precision oncology, particularly in liver cancer treatment. AI-driven techniques enhance the development of nanoparticles, optimizing their design, functionality, and targeting mechanisms for improved therapeutic efficacy and minimized toxicity. Emerging studies have highlighted the success of AI in refining nanotechnology applications for targeted cancer treatments [72].

A notable case study by Chen et al. [89] demonstrated the potential of AI in designing mesoporous silica nanoparticles (MSNs) loaded with cisplatin. These nanoparticles were developed using AI algorithms trained to predict optimal pore sizes, surface functionalization, and drug release profiles to enhance tumor-specific delivery. In preclinical trials involving HCC mouse models, these AI-designed MSNs achieved a 60% reduction in tumor size compared to conventional formulations. This success was attributed to the AI's ability to fine-tune the particle’s characteristics, ensuring enhanced cellular uptake and controlled drug release directly within the tumor microenvironment [73].

Similarly, ML has proven invaluable in optimizing lipid nanoparticle formulations. One significant example involves nanoparticles designed to target KRAS mutations-common drivers in various cancers, including liver cancer. AI algorithms processed extensive datasets on KRAS mutation pathways, lipid compositions, and patient tumor profiles. As a result, the lipid nanoparticles achieved heightened specificity for KRAS-mutated cells while reducing off-target effects. Preclinical trials reported significant improvements in therapeutic outcomes, with reduced systemic toxicity and enhanced tumor suppression. The study showcased how AI could identify novel combinations of lipids and surface ligands that traditional experimental approaches might overlook. These advancements underscore the transformative impact of AI in precision oncology. By integrating vast datasets, identifying complex patterns, and predicting optimal nanoparticle designs, AI has accelerated the translation of nanotechnology from the bench to the bedside. Future research is expected to explore additional applications, such as real-time monitoring of nanoparticle biodistribution and adaptive therapy adjustments, further solidifying the role of AI in advancing precision oncology [74, 75].

## Role of AI in determining regulated cell death

Tumor nanomedicine focuses on inducing regulated cell death (RCD), such as apoptosis, ferroptosis, and necroptosis. AI assists in this by analyzing complex datasets to determine nanoparticle-mediated RCD pathways:

### Ferroptosis induction

Iron oxide nanoparticles designed using AI have been shown to trigger ferroptosis in liver cancer cells by increasing reactive oxygen species (ROS) production and lipid peroxidation. A study by Wang et al. (2023) demonstrated 40% higher tumor inhibition in ferroptosis-induced models compared to traditional treatments. The ferroptosis Induction via Iron Oxide Nanoparticles. Ferroptosis, a form of RCD distinct from apoptosis and necrosis, is characterized by iron-dependent lipid peroxidation and accumulation of reactive oxygen species (ROS). Inducing ferroptosis in cancer cells is a promising strategy for overcoming resistance to conventional therapies. AI-driven approaches have significantly advanced the design and application of nanoparticles to trigger ferroptosis in liver cancer cells. AI algorithms analyze cellular datasets to identify pathways most susceptible to ferroptosis induction. Using this data, researchers can design nanoparticles that specifically exploit these pathways [76, 77]. The case study by Wang et al. (2023). In this study, AI was used to design iron oxide nanoparticles coated with a hepatocyte-targeting peptide. These nanoparticles exhibited a 40% higher tumor inhibition rate in liver cancer models compared to conventional treatments. The AI-optimized design enhanced ROS production and lipid peroxidation within tumor cells, effectively triggering ferroptosis. Additionally, the study demonstrated reduced systemic toxicity, highlighting the precision of AI-guided nanoparticle engineering. Many liver tumors develop resistance to chemotherapy and radiation therapy. AI-designed ferroptosis-inducing nanoparticles offer a novel approach to circumvent these challenges by exploiting vulnerabilities in cancer metabolism and oxidative stress pathways [78, 79].

### Apoptosis activation

AI identifies nanoparticle formulations that effectively activate caspase-3 and −9 pathways, ensuring selective apoptosis in HCC cells. For example, nanoparticles loaded with curcumin, an apoptosis inducer, were optimized using ML to achieve higher caspase activation rates. Regulated cell death (RCD) is a fundamental mechanism by which cancer therapies induce tumor regression. Tumor nanomedicine, particularly in liver cancer, has increasingly focused on leveraging RCD pathways, including apoptosis, ferroptosis, and necroptosis [80]. AI plays a transformative role by analyzing complex datasets, optimizing nanoparticle design, and predicting interactions that can trigger these cell death mechanisms with greater specificity and efficacy. Below, we delve into how AI enhances nanoparticle-mediated RCD pathways in liver cancer treatment [81]. Apoptosis, or programmed cell death, is a highly regulated process involving the activation of caspase enzymes that dismantle cellular components in a controlled manner. While inducing apoptosis is a key goal of many cancer therapies, achieving selective activation in tumor cells without affecting normal tissues remains a challenge [82]. AI addresses this by optimizing nanoparticle formulations to enhance apoptotic specificity and efficacy. AI models analyze high-throughput screening data to identify nanoparticle formulations that can effectively activate caspase-3 and caspase-9, the key mediators of apoptosis. Curcumin, a natural compound with apoptosis-inducing properties, has limited clinical application due to poor bioavailability. AI-driven optimization of curcumin-loaded nanoparticles has significantly improved its therapeutic potential. For example, ML algorithms were used to optimize the size, charge, and surface functionalization of curcumin-loaded nanoparticles [83]. This optimization ensured targeted delivery to HCC cells and increased cellular uptake. AI-guided formulations showed higher caspase-3 and −9 activation rates, leading to selective apoptosis in liver cancer cells. Preclinical studies reported a 50% increase in tumor regression compared to non-optimized formulations, demonstrating the power of AI in enhancing therapeutic efficacy [84]. AI is also instrumental in designing dual-function nanoparticles that combine apoptosis induction with other therapeutic mechanisms, such as immune modulation or ROS generation. These systems offer a synergistic approach to liver cancer treatment, improving outcomes while reducing treatment resistance. While apoptosis and ferroptosis have been extensively studied, necroptosis a form of programmed necrosis represents another promising target for liver cancer therapy [85]. AI-driven research is beginning to explore how nanoparticles can trigger necroptosis in tumor cells. AI algorithms analyze proteomic data to identify key regulators of necroptosis, such as receptor-interacting protein kinases (RIPK1 and RIPK3). Using this data, researchers can design nanoparticles that specifically activate necroptotic pathways in liver cancer cells. AI is also exploring how nanoparticles can induce multiple RCD pathways simultaneously, such as combining ferroptosis and necroptosis triggers [86]. These synergistic approaches are particularly effective in heterogeneous tumors like HCC, where a single pathway may not be sufficient for comprehensive tumor eradication. AI enables predictive modeling of nanoparticle interactions with cancer cells, reducing the need for extensive trial-and-error experiments. This accelerates the development of effective treatments. AI can integrate patient-specific data, such as genetic mutations and tumor microenvironment characteristics, to design nanoparticles tailored to individual tumors. By optimizing nanoparticle properties, AI minimizes off-target effects, enhancing treatment safety and patient outcomes [87, 88].

## Addressing safety concerns using AI

The translation of nanotechnology into clinical practice faces significant safety challenges, including toxicity, immunogenicity, and long-term stability. AI mitigates these concerns through:

### Toxicity prediction

AI algorithms trained on datasets of nanoparticle toxicity provide insights into potential adverse effects. For example, an ML model accurately predicted the cytotoxicity of over 200 nanoparticle formulations with 92% precision. The integration of nanotechnology into clinical practice offers transformative potential in medicine, particularly for complex diseases such as cancer [89]. However, this transition is fraught with significant safety concerns that must be addressed to ensure the efficacy and acceptability of these innovations. Toxicity, immunogenicity, and the long-term stability of nanoparticles remain critical hurdles. AI has emerged as a powerful tool for mitigating these challenges, offering advanced predictive capabilities, optimization techniques, and real-time monitoring solutions [90]. Below is an in-depth exploration of how AI addresses safety concerns associated with nanotechnology. The cytotoxicity of nanoparticles is a primary concern in nanomedicine. Variability in nanoparticle composition, size, shape, and surface chemistry can lead to unintended interactions with biological systems, resulting in adverse effects such as organ damage or systemic toxicity [91]. AI algorithms, particularly ML models, play a pivotal role in predicting and mitigating these risks. AI algorithms trained on extensive datasets containing information about nanoparticle toxicity, including experimental and clinical data, allow for accurate predictions of potential adverse effects [92]. For example case study by Liu et al. [39] A ML model was trained using a dataset of over 200 nanoparticle formulations, including their physicochemical properties and cytotoxicity profiles. The model demonstrated a remarkable 92% predictive accuracy in determining the cytotoxic potential of various nanoparticles. This capability enables researchers to screen formulations computationally before advancing to costly and time-consuming experimental studies, reducing the likelihood of introducing harmful nanoparticles into preclinical or clinical phases [93].

### Minimizing immune responses with AI-optimized nanoparticle coating

The immunogenicity of nanoparticles and their ability to trigger immune responses poses another significant challenge. The immune system often recognizes nanoparticles as foreign entities, leading to rapid clearance from the bloodstream, reduced therapeutic efficacy, and potential hypersensitivity reactions. AI-driven optimization of nanoparticle coatings offers solutions to these issues [94]. PEGylation, the process of coating nanoparticles with polyethylene glycol (PEG), is a well-established method for reducing immunogenicity and prolonging circulation time. AI algorithms have been instrumental in optimizing the PEGylation process. AI simulations determine the optimal thickness and density of PEG coatings to achieve maximum stealth properties while preserving nanoparticle functionality. Beyond PEG, AI has identified other biocompatible materials, such as zwitterionic polymers, that reduce immune recognition. By analyzing immune response datasets, AI suggests alternatives that outperform traditional coatings in specific clinical scenarios. For nanoparticles targeting HCC, AI-driven designs have resulted in PEGylated liposomes with enhanced pharmacokinetics and minimal immune activation. These nanoparticles achieved prolonged systemic circulation and higher tumor accumulation, addressing key safety and efficacy concerns [95, 96].

### Real-time monitoring of nanoparticles in vivo

A critical challenge in nanotechnology is ensuring the stability and safety of nanoparticles after administration. In vivo, degradation or unexpected interactions with biological systems can lead to toxicity or reduced therapeutic efficacy. AI-integrated nanosensors and monitoring systems provide a real-time solution to this problem. Nanosensors embedded within therapeutic nanoparticles allow continuous monitoring of their behavior in the body. AI systems analyze sensor data to detect early signs of adverse reactions. For example, if a nanoparticle begins to degrade prematurely or accumulates in non-target organs, the AI system can flag these events, enabling timely interventions [97]. Using real-time data, AI models can recommend adaptive modifications to treatment protocols. For instance, adjusting the dosing schedule or introducing secondary agents to counteract observed toxicity. Gold nanoparticles functionalized with nanosensors for liver cancer imaging and therapy have been enhanced with AI algorithms to monitor ROS levels. This monitoring ensures that nanoparticles induce therapeutic effects, such as tumor cell ferroptosis, without crossing toxicity thresholds [98]. The long-term stability of nanoparticles, including their physical integrity and functional properties, is essential for ensuring safety and efficacy in clinical applications. AI facilitates the design of nanoparticles with enhanced stability. AI models analyze data on nanoparticle degradation under various physiological conditions to predict their stability over time. Factors such as temperature sensitivity, pH-dependent changes, and enzymatic degradation are considered in these predictions [99]. AI suggests optimal materials for nanoparticle construction, such as biodegradable polymers with controlled degradation rates. This ensures that nanoparticles remain stable during circulation but degrade predictably after delivering their therapeutic payload. AI plays a transformative role in clinical decision-making by integrating complex multimodal data including imaging, genomics, and proteomics, and offering actionable insights. In the realm of nanotechnology for cancer treatment, AI empowers clinicians to monitor therapeutic progress, optimize protocols, and personalize treatments based on patient-specific responses [100]. Below is an in-depth discussion of how AI facilitates therapeutic monitoring and adaptive protocols, supported by novel studies and clinical examples. AI significantly enhances the monitoring of nanoparticle-based therapies by analyzing real-time data generated from imaging and biosensing platforms. This capability is critical for assessing the effectiveness of treatment and enabling timely adjustments to therapeutic strategies. Nanoparticles designed for dual imaging and drug delivery provide simultaneous therapeutic and diagnostic (theranostic) capabilities [101]. These systems generate data about drug release, tumor targeting, and treatment response, which AI algorithms analyze to deliver actionable insights. For example, A novel study by Zhao et al. [108] demonstrated the use of AI to process imaging data from gold nanoparticles functionalized with fluorescent markers and chemotherapeutic agents. The nanoparticles allowed real-time tracking of drug release and tumor uptake in liver cancer models [102]. AI algorithms identified patterns of suboptimal drug delivery, leading to protocol adjustments that increased tumor suppression rates by 35%. AI leverages data from advanced imaging modalities, such as MRI, CT, and positron emission tomography (PET), to assess tumor response. For example, researchers have developed AI-driven nanosystems equipped with biosensors that detect reactive oxygen species (ROS) levels in tumors. These systems provided critical feedback on the efficacy of ROS-inducing nanoparticles, enabling clinicians to determine whether the treatment was effective or required modification [103]. A study published in Nature Medicine (2021) highlighted an AI-powered system that adjusted nanoparticle-based chemotherapy regimens for HCC. The system used patient-specific data, such as liver enzyme levels and imaging results, to predict the optimal dose. Adaptive dosing reduced treatment-related toxicity by 30% compared to standard protocols while maintaining therapeutic efficacy. AI platforms enable real-time feedback loops, allowing clinicians to refine treatment strategies based on evolving patient data. An example is in a clinical trial, lipid nanoparticles designed for RNA-based therapies were combined with AI algorithms that analyzed patients’ genetic profiles and immune responses. The system dynamically adjusted the nanoparticle's lipid composition to improve delivery efficiency and reduce off-target effects. This personalized approach resulted in better therapeutic outcomes, including a 25% increase in RNA delivery efficacy [104, 105]. Table 2 highlights the applications of AI in addressing safety concerns associated with nanotechnology for liver cancer, while ensuring clinical relevance and patient-specific optimization.

**Table 2:** Applications of AI in addressing safety concerns associated with nanotechnology for liver cancer

Aspect	Key points	References
5.1 Toxicity prediction	- AI-trained algorithms predict nanoparticle cytotoxicity with high accuracy—A machine learning (ML) model achieved 92% precision in predicting cytotoxicity for over 200 nanoparticle formulations	[89–93]
	- Enables computational screening, reducing costs and risks before experimental trials	
	Case Study: ML model trained on nanoparticle datasets predicted potential adverse effects, aiding safe preclinical development	
5.2 Minimizing immune responses	- AI optimizes nanoparticle coatings (e.g., PEGylation) to reduce immunogenicity and extend circulation time	[94–96]
	- Identifies alternative coatings (e.g., zwitterionic polymers) that enhance stealth properties and functionality	
	Example: AI-designed PEGylated liposomes for HCC exhibited prolonged systemic circulation, reduced immune activation, and improved tumor targeting	
5.3 Real-time monitoring in vivo	- AI-integrated nanosensors monitor nanoparticle behavior, detecting toxicity and ensuring stability post-administration	[97–99]
	- Adaptive protocols: AI adjusts dosing or introduces counteragents based on real-time monitoring data	
	Example: Gold nanoparticles functionalized with nanosensors monitored reactive oxygen species (ROS) levels in vivo, ensuring therapeutic efficacy without exceeding toxicity thresholds	
Long-term stability and degradation	- AI predicts nanoparticle stability under physiological conditions, analyzing factors like pH sensitivity, temperature, and enzymatic degradation	[100]
	- Suggests materials (e.g., biodegradable polymers) with controlled degradation rates, enhancing safety and efficacy	
Clinical decision-making	- AI integrates multimodal data (e.g., imaging, genomics, proteomics) to optimize treatment protocols and monitor therapeutic progress	[101–105]
	- AI-driven nanosystems analyze tumor response through dual imaging and drug delivery platforms (theranostics)	
	Example: AI-enhanced lipid nanoparticles dynamically adjusted lipid composition based on genetic profiles and immune responses, improving RNA delivery by 25% and reducing off-target effects	
Adaptive treatment strategies	- AI-powered systems refine chemotherapy regimens using patient-specific data (e.g., liver enzyme levels, imaging results)	[104, 105]
	Case study: AI-adaptive dosing for HCC reduced treatment-related toxicity by 30% while maintaining therapeutic efficacy	

## Regulatory and ethical concerns in AI and nanomedicine

The intersection of AI and nanotechnology presents unique regulatory and ethical challenges, particularly in the context of liver cancer treatment. Unlike traditional pharmaceuticals, nanomedicine and AI-driven technologies require new frameworks for evaluation, approval, and monitoring. One of the primary challenges in nanomedicine is the absence of well-defined regulatory frameworks that can comprehensively address the complexities of AI-nanotechnology-based treatments. Regulatory agencies, such as the U.S. Food and Drug Administration (FDA) and the European Medicines Agency (EMA), have well-established procedures for evaluating traditional drugs, but these frameworks are insufficient for advanced therapies. Nanoparticles, for instance, vary in size, shape, surface charge, and composition, which can significantly affect their safety, efficacy, and pharmacokinetics [106]. AI-based algorithms introduce another layer of complexity, as they require real-time data processing and decision-making, which can vary from patient to patient. Regulatory agencies need to establish clear guidelines for the approval of AI-driven nanomedicine, which can consider both the material properties of nanoparticles and the computational logic of AI algorithms. This may involve the use of adaptive clinical trial designs, where AI-driven nanomedicine platforms are continuously monitored and optimized as new data is collected [107]. However, such frameworks are still in their infancy, creating uncertainty for developers and clinicians seeking to bring these treatments to market. Ethical concerns also arise in the deployment of AI in liver cancer treatment. One significant issue is the transparency of AI algorithms. Most AI systems, particularly those based on ML, function as "black boxes" where the decision-making process is not easily explainable. In a clinical setting, it is crucial that physicians and patients understand how AI recommendations are made, particularly when high-stakes decisions, such as cancer treatment plans, are involved. Ensuring the explainability and accountability of AI systems remains a key ethical challenge. Another ethical concern is the potential for AI to exacerbate existing healthcare disparities [108].

This is particularly concerning in liver cancer, which has varying incidence and outcomes based on geographical, ethnic, and socioeconomic factors. As AI takes on a more prominent role in decision-making, questions arise about patient autonomy and informed consent. AI systems can recommend personalized treatment plans based on complex data analysis that patients and even physicians may not fully understand. Ensuring that patients provide informed consent for AI-based treatments, particularly when they involve novel nanomedicines, requires a reevaluation of the current consent processes [109].

## Challenges and future directions

### The personalized nature of AI-guided nanoparticle designs

It poses challenges for large-scale production. Patient-specific nanosystems, optimized for unique genomic or proteomic profiles, require highly customized formulations. This complexity can slow down production and increase costs, issues making it difficult to scale the technology for broader clinical applications. The solution is that AI has the potential to streamline manufacturing by identifying production bottlenecks and proposing standardized yet adaptable protocols. For example, generative adversarial networks (GANs) can predict nanoparticle configurations that balance personalization with manufacturability [110]. Additionally, AI can model production workflows, optimizing the allocation of resources such as raw materials and quality control checkpoints. The prospects Integrating AI with robotic automation could further enhance the efficiency of nanoparticle manufacturing, allowing for real-time adjustments during production to meet personalized design specifications without compromising scalability. *The regulatory hurdle* is AI can assist in designing clinical trial protocols by simulating outcomes and expediting regulatory approval [111]. The regulatory landscape for nanomedicine is complex, given the novelty and variability of AI-designed nanoparticles. The issue is that the lack of standardized frameworks for evaluating the safety and efficacy of AI-driven nanosystems slows down clinical translation. Traditional regulatory pathways often do not accommodate the dynamic and predictive nature of AI-based approaches. The AI Solution is that AI can support the regulatory process by simulating clinical trial outcomes and optimizing trial designs. For instance, ML models can analyze preclinical and early-phase clinical data to predict long-term outcomes, reducing the need for extensive and costly trials. AI can also assist in identifying surrogate markers for efficacy and safety, expediting approval processes. For example in silico trials powered by AI, which simulate patient responses to nanoparticle therapies, have shown promise in reducing the time and cost associated with regulatory approvals while ensuring compliance with safety standards [112, 113]. *In the case of integration of multimodal data,* the AI should integrate imaging, genomic, and clinical data to refine nanoparticle design further. A key challenge in optimizing nanoparticle designs is the integration of diverse datasets, including imaging, genomic, and clinical data. The issue is that these datasets are often complex, high-dimensional, and collected from different platforms, making integration difficult. Without effective data fusion, critical insights into tumor biology and patient-specific needs may be missed. The AI Solution is Advanced AI algorithms, such as deep learning and multi-omics integration models, which can synthesize data from various sources [114]. One of the primary challenges in scaling nanotechnology for liver cancer treatment is the complexity of nanoparticle synthesis. The production of nanoparticles with consistent size, shape, surface characteristics, and drug loading requires precise control over multiple variables. Small deviations during the manufacturing process can lead to significant variations in the final product, affecting its safety and efficacy. Developing scalable, reproducible manufacturing processes is essential for the widespread clinical use of nanotechnology. Nanomedicine therapies require novel clinical trial designs that account for the unique characteristics of nanoparticles [115]. Traditional clinical trials often assess the safety and efficacy of small molecules or biologics, but nanoparticles behave differently in the body. Their biodistribution, cellular uptake, and clearance mechanisms differ from conventional drugs, necessitating the development of new trial protocols. Additionally, regulatory agencies may require additional preclinical data to assess the long-term effects of nanoparticles, particularly their potential to accumulate in organs such as the liver. The high cost of developing and manufacturing nanomedicines is another barrier to their clinical implementation. Nanoparticle-based therapies often involve complex, multi-step production processes that are more expensive than traditional drug manufacturing. This cost can make nanomedicine treatments inaccessible to patients in low- and middle-income countries, exacerbating global health disparities [116]. Another example is the use of AI in improving the diagnostic capabilities of nanotechnology-based imaging systems. Nanoparticles are often used as contrast agents in imaging techniques such as MRI and PET (positron emission tomography), enhancing the visibility of liver tumors. AI algorithms have been integrated into imaging platforms to analyze the distribution and localization of nanoparticles, improving the accuracy of tumor detection. A study by Kang and colleagues [38] demonstrated the use of AI-driven image analysis to improve the sensitivity and specificity of MRI in liver cancer diagnosis. This not only allowed for earlier detection of small tumors but also differentiated between malignant and benign lesions, reducing unnecessary biopsies and treatments [117, 118]. Additionally, AI has been employed in the development of nanoparticle-based gene therapies for liver cancer. Gene therapy, which involves the introduction of genetic material into cells to fight disease, has been enhanced by using nanotechnology to deliver the genetic payload to cancer cells. AI algorithms have been used to model and optimize the release kinetics of these nanoparticle systems, ensuring the timely and controlled delivery of therapeutic genes. This has resulted in improved therapeutic outcomes, as demonstrated by Wang et al. (2021), who used AI-driven models to fine-tune the release profiles of nanoparticles loaded with tumor-suppressing genes for HCC treatment [119].

### AI-driven optimization of nanocarrier design

One of the most significant advantages of AI in nanotechnology is its ability to optimize the design of nanocarriers for liver cancer treatment. Traditional methods of designing nanocarriers often involve trial and error, which can be time-consuming and costly. AI, particularly ML, can analyze large datasets of nanoparticle properties, drug release profiles, and therapeutic outcomes to predict the best design parameters for specific applications. Nanocarrier design involves several critical factors, including size, shape, surface charge, and the choice of materials used in constructing the nanoparticle [119]. Each of these parameters affects the biodistribution, cellular uptake, and drug release profile of the nanocarrier. For instance, spherical nanoparticles are known to have better circulation times in the bloodstream compared to rod-shaped ones, but rod-shaped particles may penetrate tumors more effectively. AI algorithms can analyze such relationships and predict the ideal shape for a given therapeutic goal. AI can also optimize the surface functionalization of nanocarriers. Surface functionalization, such as the attachment of targeting ligands or polyethylene glycol (PEG) to nanoparticles, enhances their ability to target liver cancer cells specifically while avoiding healthy tissues [120]. AI models can predict the most effective ligand-receptor interactions, increasing the chances of successful targeting and drug delivery. For example, AI has been used to model interactions between liver cancer cell surface receptors and various nanoparticle surface ligands, optimizing the nanoparticle design for targeted drug delivery to HCC cells. In addition to optimizing the physical characteristics of nanocarriers, AI also plays a crucial role in optimizing the drug release kinetics. Drug release from nanocarriers is typically designed to occur in response to specific triggers, such as changes in pH or temperature. AI models can simulate how different release mechanisms will perform in vivo, allowing for more precise control of drug release profiles. In liver cancer treatment, where the tumor microenvironment is often acidic, AI can help design nanocarriers that release their payload specifically in this environment, maximizing the therapeutic effect while minimizing systemic side effects [121].

### Role of AI in monitoring drug release and efficacy

Monitoring drug release and efficacy is critical in cancer treatment, where precise control over drug dosing and timing can significantly impact patient outcomes. AI algorithms have been integrated into nanotechnology platforms to monitor drug release in real-time and adjust dosing regimens based on the patient’s response. Nanoparticles are often engineered to release their drug payload over a sustained period, ensuring a steady therapeutic effect. However, variations in the tumor microenvironment or patient physiology can alter the drug release kinetics, potentially leading to underdosing or overdosing. AI systems can monitor these changes by analyzing real-time data from biosensors or imaging modalities integrated with the nanocarrier. This real-time feedback allows for adjustments in the treatment plan, ensuring optimal therapeutic efficacy [122]. In a study by Kim et al. [38], AI was used to monitor the release of doxorubicin, a chemotherapeutic agent, from nanoparticles designed for liver cancer treatment. The AI system analyzed data from biosensors embedded in the nanocarriers, detecting changes in pH and temperature that indicated drug release. The system then adjusted the drug release rate in response to these changes, ensuring that the drug was delivered at the optimal concentration to the tumor site [123].

### Enhancing precision medicine through nanotechnology and AI collaboration

Precision medicine aims to tailor treatments to individual patients based on their unique genetic, molecular, and environmental factors. The combination of nanotechnology and AI is perfectly suited to enhance precision medicine, particularly in complex diseases like liver cancer. Nanotechnology enables the precise delivery of therapeutics to the tumor site, while AI provides the computational power to analyze patient-specific data and optimize treatment plans. AI's ability to integrate large datasets, such as genomic data, proteomic profiles, and patient histories, is essential for developing personalized treatment regimens [124]. For example, a patient’s genetic profile might indicate that they are more likely to respond to a particular chemotherapy drug or immunotherapy. AI can analyze this information and suggest a treatment plan that includes the optimal nanocarrier design and drug release profile for that individual patient. Nanotechnology further enhances this precision by allowing for the targeted delivery of drugs, ensuring that they reach the tumor cells with minimal impact on healthy tissues. For liver cancer, where underlying liver conditions such as cirrhosis or hepatitis can complicate treatment, this level of precision is particularly important [125]. One notable case study is the development of AI-assisted nanocarriers for precision medicine in liver cancer by Huang et al. [35]. Their study utilized AI to analyze a patient’s tumor genetic profile and predict the best combination of drugs to deliver via nanocarriers. This personalized approach led to improved therapeutic outcomes, with fewer side effects compared to standard treatment protocols. In the context of clinical trials, AI can also identify patients who are most likely to benefit from nanotechnology-based treatments. By analyzing patient-specific biomarkers and genetic mutations, AI algorithms can predict which patients are good candidates for clinical trials involving nanoparticle-based therapies. This increases the likelihood of successful outcomes and accelerates the development of new liver cancer treatments [126].

### Limitations of current AI algorithms in liver cancer treatment

AI holds great promise in liver cancer treatment, but current algorithms have several limitations that must be addressed before they can be fully integrated into clinical practice. AI algorithms rely on large, high-quality datasets to make accurate predictions. However, in the case of liver cancer, data is often limited, incomplete, or biased. Many datasets used to train AI models are based on Western populations, which may not generalize well to patients in other regions, such as Asia, where liver cancer is more prevalent. Additionally, medical imaging data can be noisy or inconsistent, affecting the performance of AI models in detecting and diagnosing liver cancer. Another limitation of current AI algorithms is their tendency to overfit training data [127]. Overfitting occurs when an AI model performs well on the data it was trained on but fails to generalize to new, unseen data. In the context of liver cancer treatment, this means that an AI algorithm may work well in a controlled research setting but perform poorly in real-world clinical environments. Overfitting can be mitigated by using techniques such as cross- validation and regularization, but ensuring that AI models generalize well remains a key challenge. AI algorithms must be integrated seamlessly into existing clinical workflows to be effective. However, many current AI tools are not designed with clinicians in mind. These tools may require extensive training to use, and their recommendations may not be easily interpretable by healthcare professionals. Ensuring that AI systems are user-friendly and provide actionable insights is critical for their adoption in clinical practice [128].

## Conclusion

The convergence of nanotechnology and AI represents a groundbreaking approach to tackling liver cancer. Nanotechnology enables precise drug delivery and enhanced therapeutic outcomes, while AI offers powerful tools for early detection, diagnosis, and personalized treatment strategies. Together, these technologies have the potential to revolutionize liver cancer treatment, improving patient outcomes and making therapies more accessible.

However, several challenges remain before this potential can be fully realized. Regulatory, ethical, and data privacy concerns must be addressed, while limitations in AI algorithms—such as data bias, overfitting, and integration into clinical workflows—require significant attention. Moreover, large-scale data sharing and the development of high-quality, diverse datasets are crucial to ensuring the accuracy and effectiveness of AI models in liver cancer treatment.

By overcoming these challenges through interdisciplinary collaboration between researchers, clinicians, and regulatory bodies, the full promise of AI-enhanced nanomedicine can be unlocked. This will lead to more effective, personalized, and scalable solutions for liver cancer, transforming the landscape of cancer treatment in the years to come [129, 130].

## Data Availability

No datasets were generated or analysed during the current study.

## References

[1] Ambros V. microRNAs: tiny regulators with great potential. Cell. 2001;107(7):823–6. 11779458 10.1016/s0092-8674(01)00616-x

[2] Boldrini L, Bibault JE, Masciocchi C, Shen Y, Bittner MI. Deep learning: a review for the radiation oncologist. Front Oncol. 2020;9:977. 10.3389/fonc.2019.00977 PMC6779810 31632910

[3] Bruix J, Sherman M. Management of hepatocellular carcinoma: an update. Hepatology. 2011;53(3):1020–2. 21374666 10.1002/hep.24199 PMC3084991

[4] Cheng X, Cheng L, Dai H. Artificial intelligence-based diagnostic models for hepatocellular carcinoma: a review. Cancer Manag Res. 2020;12:11181–7.

[5] Dobrovolskaia MA, McNeil SE. Immunological properties of engineered nanomaterials. Nat Nanotechnol. 2007;2(8):469–78. 18654343 10.1038/nnano.2007.223

[6] El-Serag HB. Hepatocellular carcinoma. N Engl J Med. 2011;365(12):1118–27. 21992124 10.1056/NEJMra1001683

[7] Etheridge ML, Campbell SA, Erdman AG, Haynes CL, Wolf SM, McCullough J. The big picture on nanomedicine: The state of investigational and approved nanomedicine products. Nanomed Nanotech Biol Med. 2013;9(1):1–14. 10.1016/j.nano.2012.05.013 PMC4467093 22684017

[8] Etheridge ML, Campbell SA, Erdman AG, Haynes CL, Wolf SM, McCullough J. The big picture on nanomedicine: the state of investigational and approved nanomedicine products. Nanomed Nanotechnol Biol Med. 2013;9(1):1–14. 10.1016/j.nano.2012.05.013 PMC4467093 22684017

[9] Banerjee R, Gupta N, Yadav N, Ponnusamy MP. Sorafenib-loaded lipid nanoparticles enhance therapeutic efficacy in liver cancer: in vitro and in vivo studies. Nanomed Nanotechnol Biol Med. 2019;16(1):235–45.

[10] Barenholz Y. Doxil®—the first FDA-approved nano-drug: lessons learned. J Control Release. 2012;160(2):117–34. 22484195 10.1016/j.jconrel.2012.03.020

[11] Danhier F, Ansorena E, Silva JM, Coco R, Le Breton A, Préat V. PLGA-based nanoparticles: an overview of biomedical applications. J Control Release. 2012;161(2):505–22. 22353619 10.1016/j.jconrel.2012.01.043

[12] Dandawate P, Vyas A, Ahmad A, Sarkar FH. Nanotechnology for enhanced delivery of chemotherapeutic agents in cancer therapy. Pharmacol Ther. 2012;136(3):235–46.

[13] Garud A, Singh D, Garud N. Solid lipid nanoparticles (SLN): method, characterization, and applications. Int Curr Pharmaceutical J. 2012;1(11):384–93.

[14] Gurunathan S, Han JW, Dayem AA, Eppakayala V, Kim JH. Oxidative stress-mediated antibacterial activity of graphene oxide and reduced graphene oxide in *Pseudomonas aeruginosa*. Int J Nanomed. 2014;9(1):2051–70. 10.2147/IJN.S37397 PMC3514835 23226696

[15] Leamon CP, Low PS. Folate-mediated targeting: From diagnostics to drug and gene delivery. Drug Discovery Today. 2001;6(1):44–51. 11165172 10.1016/s1359-6446(00)01594-4

[16] Qian ZM, Li H, Sun H, Ho K. Targeted drug delivery via the transferrin receptor-mediated endocytosis pathway. Pharmacol Rev. 2002;54(4):561–87. 12429868 10.1124/pr.54.4.561

[17] Sun T, Zhang YS, Pang B, Hyun DC, Yang M, Xia Y. Engineered nanoparticles for drug delivery in cancer therapy. Angew Chem Int Ed. 2014;53(46):12320–64. 10.1002/anie.201403036 25294565

[18] Wang-Gillam A, Li CP, Bodoky G, Dean A, Shan YS, Jameson G, et al. Nanoliposomal irinotecan with fluorouracil and folinic acid in metastatic pancreatic cancer after previous gemcitabine-based therapy (NAPOLI-1): a global, randomized, open-label, phase 3 trial. Lancet. 2016;387(10018):545–57. 26615328 10.1016/S0140-6736(15)00986-1

[19] Jiang T, Zhuang J, Duan X, Liu Y, Huang Y. Gold nanoparticles- enhanced photothermal therapy: effects of surface chemistry on the treatment of cancer stem-like cells. ACS Nano. 2020;14(1):1196–211.31904217

[20] Aerts HJ, Velazquez ER, Leijenaar RT, Parmar C, Grossmann P, Carvalho S, et al. Decoding tumor phenotype by noninvasive imaging using a quantitative radiomics approach. Nat Commun. 2014;5(1):1–9. 10.1038/ncomms5006 PMC4059926 24892406

[21] Chakraborty S, Hossain MS, Islam MZ. Applications of AI in clinical trials: a literature review. Comput Biol Med. 2020;124: 103844.

[22] Dai W, Li Y, Shi J, Wang Z, Meng Q, Jin C. Prediction of hepatocellular carcinoma prognosis based on machine learning of mRNA expression data. EBioMedicine. 2020;54: 102710.32283530

[23] Esteva A, Kuprel B, Novoa RA, Ko J, Swetter SM, Blau HM, Thrun S. Dermatologist-level classification of skin cancer with deep neural networks. Nature. 2017;542(7639):115–8. 28117445 10.1038/nature21056 PMC8382232

[24] Hagner O, Ward T, Bruggeman K, Hanson R. AI and robotic surgery: future directions for precision oncology. J Robot Surg. 2021;15(3):345–52.

[25] Hamm CA, Wang A, Robbins LA, Eschelman DJ, Gonsalves CF, Li S. Deep learning for liver cancer detection. IEEE Trans Med Imaging. 2019;38(6):1429–38.

[26] Kalpathy-Cramer J, Mamom G, Singh R. Radiomics in precision oncology: from imaging to clinical practice. Radiology. 2017;285(2):663–74.

[27] King DR, Chambers AM, Lim JH. AI in robotic-assisted liver surgery. Surgical Innovation. 2019;26(3):210–21.

[28] Liang C, Huang Y, Yu M, Li R, Zeng W, Zhang L. Machine learning- based prediction of treatment outcomes for liver cancer. J Cancer Res Clin Oncol. 2018;144(12):2465–73. 30259149 10.1007/s00432-018-2756-8 PMC6244647

[29] Renz JF, Forman LM, Gardner T. AI-enhanced clinical decision support for liver transplant. Hepatology. 2019;70(5):1847–58.

[30] Topol EJ. High-performance medicine: The convergence of human and artificial intelligence. Nat Med. 2019;25(1):44–56. 30617339 10.1038/s41591-018-0300-7

[31] Wang S, Yu H, Feng Q. Personalized treatment in liver cancer using AI-based approaches. Liver Int. 2021;41(8):1986–93.

[32] Yasaka K, Akai H, Kunimatsu A, Abe O, Kiryu S. Deep learning with convolutional neural network for differentiation of liver masses at dynamic contrast-enhanced CT: a preliminary study. Radiology. 2018;286(3):887–96. 29059036 10.1148/radiol.2017170706

[33] Sun C, Xu A, Liu D, Xiong Z, Zhao F, Ding W. Deep learning-based classification of liver cancer histopathology images using only global labels. IEEE J Biomed Health Inform [Internet]. 2020;24(6):1643–51. 10.1109/jbhi.2019.2949837. 10.1109/JBHI.2019.2949837 31670686

[34] Zhu Z, Shen Y, Xu L, Liu Q. Machine learning in liver cancer: Progress and challenges. Artif Intell Med. 2021;114: 102039.33875158

[35] Huang Y, Chen M, Yuan L, Liu X, Wang H. AI-driven nanomedicine for precision liver cancer therapy. Nat Nanotechnol. 2020;15(7):512–9.32533115

[36] Jiang W, Kim BY, Rutka JT, Chan WC. Nanoparticle-mediated cellular response is size-dependent. Nat Nanotechnol. 2018;3(1):145–50. 10.1038/nnano.2008.30 18654486

[37] Karimi M, Ghasemi A, Zangabad PS, Rahighi R, Basri SMM, Mirshekari H. Smart micro/nanoparticles in stimulus-responsive drug/gene delivery systems. Chem Soc Rev. 2016;45(5):1457–501. 26776487 10.1039/c5cs00798d PMC4775468

[38] Kim SJ, Lim JY, Yoon HJ, Kang SY. AI-assisted monitoring of doxorubicin release from nanoparticles in liver cancer treatment. Adv Drug Deliv Rev. 2019;143:202–11.

[39] Liu H, Chen L, Chen H, Zhang M, Zheng J. AI-enhanced clinical trials for liver cancer: targeting precision medicine. J Hepatol. 2020;72(5):1151–9.32145255

[40] Wang H, Chen L, Xu Z, Zhang Y. AI-powered biosensors for real-time monitoring of liver cancer therapy. Biosens Bioelectron. 2020;145: 111722.

[41] Yang W, Thordarson P, Gooding JJ. Nanoparticle toxicity: the role of AI in the development of safer nanomaterials. Nat Rev Mater. 2019;4(8):567–80.

[42] Yin L, Cai W, Ma Z. AI-enhanced imaging for liver cancer detection: a nanotechnology perspective. IEEE Trans Med Imaging. 2020;39(5):1245–52.31603816

[43] Baldwin J, Jordan D, Moore A. Regulating AI-driven nanomedicine: current frameworks and future challenges. J Regul Sci. 2020;12(3):55–72.

[44] Brisimi TS, Chen R, Mela T, Olshevsky A, Paschalidis IC, Shi W. Federated learning of predictive models from federated electronic health records. Artif Intell Med. 2018;96:31–43. 10.1016/j.ijmedinf.2018.01.007 PMC5836813 29500022

[45] D’Mello SR, Cruz CN, Chen ML, Kapoor M, Lee SL, Tyner KM. The evolving landscape of drug products containing nanomaterials in the United States. Nat Nanotechnol. 2017;12(6):523–9. 28436961 10.1038/nnano.2017.67

[46] Dwork C, Roth A. The algorithmic foundations of differential privacy. Foundations Trends Theor Comput Sci. 2014;9(3–4):211–407.

[47] Floridi L, Cowls J, Beltrametti M, Chatila R, Chazerand P, Dignum V. AI4People—an ethical framework for a good AI society: Opportunities, risks, principles, and recommendations. Mind Mach. 2018;28(4):689–707. 10.1007/s11023-018-9482-5 PMC6404626 30930541

[48] González-Fernández A, Pedraz JL, Orive G. Therapeutic applications of nanotechnology in cancer treatment. Pharm Res. 2020;37(5):11–25.

[49] Hare JI, Lammers T, Ashford MB, Puri S, Storm G, Barry ST. Challenges and strategies in anti-cancer nanomedicine development: an industry perspective. Adv Drug Deliv Rev. 2017;108:25–38. 27137110 10.1016/j.addr.2016.04.025

[50] Kessler DA, Sun Y, Ehrenberg AS. Regulating nanotechnology in drug development: a decade of progress. Regul Toxicol Pharmacol. 2017;90:358–68.28870489

[51] Mittelstadt BD, Allo P, Taddeo M, Wachter S, Floridi L. The ethics of algorithms: mapping the debate. Big Data Soc. 2016;3(2):1–21.

[52] Nissenbaum H. Privacy in context: technology, policy, and the integrity of social life. Stanford University Press; 2019.

[53] Reed TR, Vasilenko SA, Hawk JA. Racial and geographic disparities in liver cancer outcomes: the role of AI-driven interventions. Cancer Epidemiol. 2020;68: 101794.32795946

[54] Rieke N, Hancox J, Li W, Milletari F, Roth HR, Albarqouni S. The future of digital health with federated learning. Nat Med. 2020;26(1):23–6. 33015372 10.1038/s41746-020-00323-1 PMC7490367

[55] Rutter P, Singh S, Fielding J. Artificial intelligence in liver cancer: an unmet need for diverse datasets. J Hepatol. 2021;75(2):507–9.

[56] Institute T. Addressing overfitting in AI: best practices for the healthcare industry. J AI Res. 2020;3(1):12–8.

[57] Ahmad T, Bustam MA, Zulfiqar M, Moniruzzaman M, Idris A, Iqbal J, Asghar HM, Ullah S. Controllable phytosynthesis of gold nanoparticles and investigation of their size and morphology-dependent photocatalytic activity under visible light. J Photochem Photobiol A. 2020;392:112429. 10.1016/j.jphotochem.2020.112429.

[58] Ahmad W, Singh A, Jaiswal KK, Gupta P. Green synthesis of photocatalytic TiO 2 nanoparticles for potential application in photochemical degradation of ornidazole. J Inorg Organomet Polym Mater. 2021;31:614–23. 10.1007/s10904-020-01703-6.

[59] Wang Y, Ding G, Lin K, Liu Y, Deng X, Li Q. Facile one-pot synthesis of ultrathin carbon layer encapsulated magnetite nanoparticle and graphene oxide nanocomposite for efficient removal of metal ions. Sep Purif Technol. 2021;266: 118550. 10.1016/j.seppur.2021.118550.

[60] Hu Q, Liu Y, Gu X, Zhao Y. Adsorption behavior and mechanism of different arsenic species on mesoporous MnFe2O4 magnetic nanoparticles. Chemosphere. 2017;181:328–36. 10.1016/j.chemosphere.2017.04.049. 28453965 10.1016/j.chemosphere.2017.04.049

[61] Lu J, Batjikh I, Hurh J, Han Y, Ali H, Mathiyalagan R, Ling C, Ahn JC, Yang DC. Photocatalytic degradation of methylene blue using biosynthesized zinc oxide nanoparticles from bark extract of Kalopanax septemlobus. Optik. 2019;182:980–5. 10.1016/j.ijleo.2018.12.016.

[62] Singh H, Du J, Singh P, Yi TH. Extracellular synthesis of silver nanoparticles by *Pseudomonas* sp. THG-LS1.4 and their antimicrobial application. J Pharm Anal. 2018;8(4):258–64. 10.1016/j.jpha.2018.04.004. 30140490 10.1016/j.jpha.2018.04.004 PMC6104148

[63] Mahlaule-Glory LM, Mathobela S, Hintsho-Mbita NC. Biosynthesized bimetallic (ZnOSnO2) nanoparticles for photocatalytic degradation of organic dyes and pharmaceutical pollutants. Catalysts. 2022;12(3):334. 10.3390/catal12030334.

[64] Shabani N, Javadi A, Jafarizadeh-Malmiri H, Mirzaie H, Sadeghi J. Potential application of iron oxide nanoparticles synthesized by co-precipitation technology as a coagulant for water treatment in settling tanks. Mining Metall Explore. 2021;38:269–76. 10.1007/s42461-020-00338-y.

[65] Ng MF, Sun Y, Seh ZW. Machine learning-inspired battery material innovation. Energy Adv. 2023;2(4):449–64. 10.1039/d3ya00040k.

[66] Zhang Z, Zhang H, Yao Y, Wang J, Guo H, Deng Y, Han X. Controlled synthesis and structure engineering of transition metal-based nanomaterials for oxygen and hydrogen electrocatalysis in zinc-air battery and water-splitting devices. Chemsuschem. 2021;14(7):1659–73. 10.1002/cssc.202002944. 33565262 10.1002/cssc.202002944

[67] Li M, Dai L, Hu Y. Machine learning for harnessing thermal energy: from materials discovery to system optimization. ACS Energy Lett. 2022;7(10):3204–26. 10.1021/acsenergylett.2c01836. 37325775 10.1021/acsenergylett.2c01836 PMC10264155

[68] Bai L, Zhang Y, Tong W, Sun L, Huang H, An Q, Tian N, Chu PK. Graphene for energy storage and conversion: synthesis and interdisciplinary applications. Electrochem Energy Rev. 2020;3(2):395–430. 10.1007/s41918-019-00042-6.

[69] Bandara TM, Aththanayake AA, Gunasekara LB, Wijesundara WM. Applications of quantum dots in energy conversion and storage devices, In: Quantum dots. 2023; pp. 383–419. 10.1016/b978-0-323-85278.

[70] Peng B, Jiang S, Zhang Y, Zhang J. Enrichment of metallic carbon nanotubes by electric field-assisted chemical vapor deposition. Carbon. 2011;49(7):2555–60. 10.1016/j.carbon.2011.02.045.

[71] Meng D, Duan H, Wu S, Ren X, Yuan S. Lithium iron phosphate with high-rate capability synthesized through hydrothermal reaction in low Li concentration solution. J Alloys Compd. 2023;967: 171570. 10.1016/j.jallcom.2023.171570.

[72] Wang C, Cai X, Chen Y, Cheng Z, Luo X, Mo S, Jia L, Lin P, Yang Z. Improved hydrogen production from glycerol photoreforming over sol-gel derived TiO2 coupled with metal oxides. Chem Eng J. 2017;317:522–32. 10.1016/j.cej.2017.02.033.

[73] Chuhadiya S, Suthar D, Patel SL, Dhaka MS. Metal-organic frameworks as hybrid porous materials for energy storage and conversion devices: a review. Coord Chem Rev. 2021;446: 214115. 10.1016/j.ccr.2021.214115.

[74] Younis A, Lin CH, Guan X, Shahrokhi S, Huang CY, Wang Y, He T, Singh S, Hu L, Retamal JR, He JH. Halide perovskites: a new era of solution-processed electronics. Adv Mater. 2021;33(23):2005000. 10.1002/adma.202005000. 10.1002/adma.202005000 33938612

[75] Zhou G, Xu L, Hu G, Mai L, Cui Y. Nanowires for electrochemical energy storage. Chem Rev. 2019;119(20):11042–109. 10.1021/acs.chemrev.9b00326. 31566351 10.1021/acs.chemrev.9b00326

[76] Upadhyay SN, Satrughna JA, Pakhira S. Recent advancements of two- dimensional transition metal dichalcogenides and their applications in electrocatalysis and energy storage. Emergent Mater. 2021;4(4):951–70. 10.1007/s42247-021-00241-2.

[77] Stecuła K, Wolniak R, Grebski WW. AI-Driven urban energy solutions—from individuals to society: a review. Energies. 2023;16(24):7988. 10.3390/en16247988.

[78] Wang S, Zhang Y, Qin Y, Lu J, Liu W. Improvement of TiN coating on comprehensive performance of NiTi alloy braided vascular stent. Ceram Int [Internet]. 2023;49(9):13405–13. 10.1016/j.ceramint.2022.12.215.

[79] Gupta N, Gupta SM, Sharma SK. Carbon nanotubes: synthesis, properties and engineering applications. Carbon Lett. 2019;29:419–47. 10.1007/s42823-019-00068-2.

[80] Yin Z, Cui C, Chen H, Duoni YuX, Qian W. The application of carbon nanotube/ graphene-based nanomaterials in wastewater treatment. Small. 2020;16(15):1902301. 10.1002/smll.201902301. 10.1002/smll.201902301 31788946

[81] Chaichi A, Wang Y, Garcia MR. Substrate engineered interconnected graphene electrodes with ultrahigh energy and power densities for energy storage applications. ACS Appl Mater Interfaces. 2018;10(25):21235–45. 10.1021/acsami.8b03020. 29856205 10.1021/acsami.8b03020

[82] Ghaly HA, El-Deen AG, Souaya ER, Allam NK. Asymmetric supercapacitors based on 3D graphene-wrapped V2O5 nanospheres and Fe3O4@ 3D graphene electrodes with high power and energy densities. Electrochim Acta. 2019;310:58–69. 10.1016/J.ELECTACTA.2019.04.071.

[83] Ahmad T, Zhang D, Huang C, Zhang H, Dai N, Song Y, Chen H. Artificial intelligence in sustainable energy industry: status Quo, challenges and opportunities. J Clean Prod. 2021;289: 125834. 10.1016/j.jclepro.2021.125834.

[84] Dong H, Lin J, Tao Y, Jia Y, Sun L, Li WJ, Sun H. AI-enhanced biomedical micro/nanorobots in microfluidics. Lab Chip. 2024;24(5):1419–40. 10.1039/d3lc00909b. 38174821 10.1039/d3lc00909b

[85] Kong X, Gao P, Wang J, Fang Y, Hwang KC. Advances of medical nanorobots for future cancer treatments. J Hematol Oncol. 2023;16(1):74. 10.1186/s13045-023-01463-z. 37452423 10.1186/s13045-023-01463-z PMC10347767

[86] Tan TZ, Quek C, Ng GS, Ng EY. A novel cognitive interpretation of breast cancer thermography with complementary learning fuzzy neural memory structure. Expert Syst Appl. 2007;33(3):652–66. 10.1016/j.eswa.2006.06.012. 32288331 10.1016/j.eswa.2006.06.012 PMC7126614

[87] Agrahari V, Agrahari V, Chou ML, Chew CH, Noll J, Burnouf T. Intelligent micro-/nanorobots as drug and cell carrier devices for biomedical therapeutic advancement: promising development opportunities and translational challenges. Biomaterials. 2020;260: 120163. 10.1016/j.biomaterials.2020.120163. 32882512 10.1016/j.biomaterials.2020.120163

[88] Nistor MT, Rusu AG. Nanorobots with applications in medicine. In: Polymeric nanomaterials in nanotherapeutics. 2019; Amsterdam: Elsevier, pp. 123–149. 10.1016/b978-0-12-813932-5.00003-0.

[89] Chen Y, Chen D, Liang S, Dai Y, Bai X, Song B, Zhang D, Chen H, Feng L. Recent advances in field-controlled micro–nano manipulations and micro–nano robots. Adv Intell Syst. 2022;4(3):2100116. 10.1002/aisy.202100116.

[90] Nosrati H, Nosrati M. Artificial intelligence in regenerative medicine: applications and implications. Biomimetics. 2023;8(5):442. 10.3390/biomimetics8050442. 37754193 10.3390/biomimetics8050442 PMC10526210

[91] Neto OP. Intelligent computational nanotechnology: the role of computational intelligence in the development of nanoscience and nanotechnology. J Comput Theor Nanosci. 2014;11(4):928–44. 10.1166/jctn.2014.3446.

[92] Alfieri A, Anantharaman SB, Zhang H, Jariwala D. Nanomaterials for quantum information science and engineering. Adv Mater. 2023;35(27):2109621. 10.1002/adma.202109621. 10.1002/adma.202109621 35139247

[93] Pulicharla MR. Hybrid quantum-classical machine learning models: powering the future of AI. J Sci Technol. 2023;4(1):40–65. 10.55662/JST.2023.4102.

[94] Shuford J. Quantum computing and artificial intelligence: synergies and challenges. JAIGS. 2024. 10.60087/jaigs.v1i1.35.

[95] Hassanzadeh P. Towards the quantum-enabled technologies for development of drugs or delivery systems. J Control Rel. 2020;324:260–79. 10.1016/j.jconrel.2020.04.050. 10.1016/j.jconrel.2020.04.050 32380203

[96] Laucht A, Hohls F, Ubbelohde N, Gonzalez-Zalba MF, Reilly DJ, Stobbe S, et al. Roadmap on quantum nanotechnologies. Nanotechnology. 2021;32(16): 162003. 10.1088/1361-6528/abb333. 33543734 10.1088/1361-6528/abb333

[97] Kumar G, Yadav S, Mukherjee A, Hassija V, Guizani M. Recent advances in quantum computing for drug discovery and development. IEEE Access. 2024. 10.1109/ACCESS.2024.3376408.

[98] Raparthi M, Gayam SR, Kasaraneni BP, Kondapaka KK, Putha S, Pattyam SP, Thuniki P, Kuna SS, Nimmagadda VS, Sahu MK. Harnessing quantum computing for drug discovery and molecular modelling in precision medicine: exploring its applications and implications for precision medicine advancement. Adv Deep Learn Tech. 2022;2(1):27–36.

[99] Luckow A, Klepsch J, Pichlmeier J. Quantum computing: towards industry reference problems. Digitale Welt. 2021;5:38–45. 10.1007/s42354-021-0335-7.

[100] El Azzaoui A, Salim MM, Park JH. Secure and reliable big-data-based decision making using quantum approach in IIoT systems. Sensors. 2023;23(10):4852. 10.3390/s23104852. 37430766 10.3390/s23104852 PMC10222651

[101] Reilly DJ. Engineering the quantum-classical interface of solid-state qubits. NPJ Quantum Info. 2015;1(1):15011. 10.1038/npjqi.2015.11.

[102] Yu CJ, Von Kugelgen S, Laorenza DW, Freedman DE. A molecular approach to quantum sensing. ACS Cent Sci. 2021;7(5):712–23. 10.1021/acscentsci.0c00737. 34079892 10.1021/acscentsci.0c00737 PMC8161477

[103] Dumke R, Lu Z, Close J, Robins N, Weis A, Mukherjee M, Birkl G, Hufnagel C, Amico L, Boshier MG, Dieckmann K. Roadmap on quantum optical systems. J Opt. 2016;18(9): 093001. 10.1088/2040-8978/18/9/093001.

[104] Hossain KA. The potential and challenges of quantum technology in modern era. Sci Res J. 2023;11(6):64–76. 10.31364/SCIRJ/v11.i6.2023.

[105] Johnson AM, Kleczko EK, Nemenoff RA. Eicosanoids in cancer: new roles in immunoregulation. Front Pharmacol. 2020;11: 595498. 33364964 10.3389/fphar.2020.595498 PMC7751756

[106] Dai E, Han L, Liu J, Xie Y, Zeh HJ, Kang R. Ferroptotic damage promotes pancreatic tumorigenesis through a TMEM173/STING-dependent DNA sensor pathway. Nat Commun. 2020;11(1):6339. 33311482 10.1038/s41467-020-20154-8 PMC7732843

[107] Sun S, Shen J, Jiang J, Wang F, Min J. Targeting ferroptosis opens new avenues for the development of novel therapeutics. Sign Transduct Target Ther. 2023;8(1):372. 10.1038/s41392-023-01606-1 PMC10514338 37735472

[108] Zhao YY, Lian JX, Lan Z, Zou KL, Wang WM, Yu GT. Ferroptosis promotes anti-tumor immune response by inducing immunogenic exposure in HNSCC. Oral Dis. 2023;29(3):933–41. 34773344 10.1111/odi.14077

[109] Dixon SJ, Patel DN, Welsch M, Skouta R, Lee ED, Hayano M. Pharmacological inhibition of cystine-glutamate exchange induces endo plasmic reticulum stress and ferroptosis. Elife. 2014;3: e02523. 24844246 10.7554/eLife.02523 PMC4054777

[110] Sun J, Wei Q, Zhou Y, Wang J, Liu Q, Xu H. A systematic analysis of FDA-approved anticancer drugs. BMC Syst Biol. 2017;11(Suppl 5):87. 28984210 10.1186/s12918-017-0464-7 PMC5629554

[111] Hu S, Ma J, Su C, Chen Y, Shu Y, Qi Z. Engineered exosome-like nanovesicles suppress tumor growth by reprogramming tumor micro environment and promoting tumor ferroptosis. Acta Biomater. 2021;135:567–81. 34506976 10.1016/j.actbio.2021.09.003

[112] Liu P, Shi X, Peng Y, Hu J, Ding J, Zhou W. Anti-PD-L1 DNAzyme loaded photothermal Mn(2+) /Fe(3+) hybrid metal-phenolic networks for cyclically amplified tumor ferroptosis-immunotherapy. Adv Healthc Mater. 2022;11(8): e2102315. 34841741 10.1002/adhm.202102315

[113] Xie L, Li J, Wang G, Sang W, Xu M, Li W. Phototheranostic metal-phenolic networks with antiexosomal PD-L1 enhanced ferroptosis for synergistic immunotherapy. J Am Chem Soc. 2022;144(2):787–97. 34985903 10.1021/jacs.1c09753

[114] Yang F, Xiao Y, Ding JH, Jin X, Ma D, Li DQ. Ferroptosis heterogeneity in triple-negative breast cancer reveals an innovative immunotherapy combination strategy. Cell Metab. 2023;35(1):84-e100108. 36257316 10.1016/j.cmet.2022.09.021

[115] Han Y, Zhang YY, Pan YQ, Zheng XJ, Liao K, Mo HY. IL-1β-associated NNT acetylation orchestrates iron-sulfur cluster maintenance and cancer immunotherapy resistance. Mol Cell. 2023;83(11):1887-e19021888. 37244254 10.1016/j.molcel.2023.05.011

[116] Fan F, Liu P, Bao R, Chen J, Zhou M, Mo Z. A dual PI3K/HDAC inhibitor induces immunogenic ferroptosis to potentiate cancer immune checkpoint therapy. Cancer Res. 2021;81(24):6233–45. 34711611 10.1158/0008-5472.CAN-21-1547

[117] Jiang Z, Lim SO, Yan M, Hsu JL, Yao J, Wei Y. TYRO3 induces anti-PD-1/PD-L1 therapy resistance by limiting innate immunity and tumoral ferroptosis. J Clin Invest. 2021;131(8): e139434. 33855973 10.1172/JCI139434 PMC8262501

[118] Li H, Sun Y, Yao Y, Ke S, Zhang N, Xiong W. USP8-governed GPX4 homeostasis orchestrates ferroptosis and cancer immunotherapy. Proc Natl Acad Sci USA. 2024;121(16): e2315541121. 38598341 10.1073/pnas.2315541121 PMC11032464

[119] Deng J, Zhou M, Liao T, Kuang W, Xia H, Yin Z. Targeting cancer cell ferroptosis to reverse immune checkpoint inhibitor therapy resistance. Front Cell Dev Biol. 2022;10: 818453. 35399527 10.3389/fcell.2022.818453 PMC8988234

[120] Meng Y, Sun HY, He Y, Zhou Q, Liu YH, Su H. BET inhibitors potentiate melanoma ferroptosis and immunotherapy through AKR1C2 inhibition. Milit Med Res. 2023;10(1):61. 10.1186/s40779-023-00497-1 PMC10694977 38049916

[121] Galon J, Bruni D. Approaches to treat immune hot, altered and cold tumours with combination immunotherapies. Nat Rev Drug Discov. 2019;18(3):197–218. 30610226 10.1038/s41573-018-0007-y

[122] Hegde PS, Chen DS. Top 10 challenges in cancer immunotherapy. Immunity. 2020;52(1):17–35. 31940268 10.1016/j.immuni.2019.12.011

[123] Binnewies M, Roberts EW, Kersten K, Chan V, Fearon DF, Merad M. Understanding the tumor immune microenvironment (TIME) for effective therapy. Nat Med. 2018;24(5):541–50. 29686425 10.1038/s41591-018-0014-x PMC5998822

[124] Srivastava MK, Sinha P, Clements VK, Rodriguez P, Ostrand-Rosenberg S. Myeloid-derived suppressor cells inhibit T-cell activation by depleting cystine and cysteine. Cancer Res. 2010;70(1):68–77. 20028852 10.1158/0008-5472.CAN-09-2587 PMC2805057

[125] Roh JL, Kim EH, Jang H, Shin D. Nrf2 inhibition reverses the resistance of cisplatin-resistant head and neck cancer cells to artesunate induced ferroptosis. Redox Biol. 2017;11:254–62. 28012440 10.1016/j.redox.2016.12.010 PMC5198738

[126] Ma S, Henson ES, Chen Y, Gibson SB. Ferroptosis is induced following siamese and lapatinib treatment of breast cancer cells. Cell Death Dis. 2016;7(7): e2307. 27441659 10.1038/cddis.2016.208 PMC4973350

[127] Kerkhove L, Geirnaert F, Dufait I, De Ridder M. Ferroptosis. Frenemy of radiotherapy. Int J Mol Sci. 2024;25(7):3641. 38612455 10.3390/ijms25073641 PMC11011408

[128] Zhu S, Zhang T, Zheng L, Liu H, Song W, Liu D. Combination strategies to maximize the benefits of cancer immunotherapy. J Hematol Oncol. 2021;14(1):156. 34579759 10.1186/s13045-021-01164-5 PMC8475356

[129] Zhang J, Shen L, Jiang Y, Sun S. Random alloy and intermetallic nanocatalysts in fuel cell reactions. Nanoscale 2020;12(38):19557–19581. 10.1039/d0nr05475e 10.1039/d0nr05475e 32986070

[130] De Leon NP, Itoh KM, Kim D, Mehta KK, Northup TE, Paik H, Palmer BS, Samarth N, Sangtawesin S, Steuerman DW. Materials challenges and opportunities for quantum computing hardware. Science 2021;372(6539):eabb2823, 10.1126/science.abb2823. 10.1126/science.abb2823 10.1126/science.abb2823 33859004

